# Expression of South East Asian Ovalocytic Band 3 Disrupts Erythroblast Cytokinesis and Reticulocyte Maturation

**DOI:** 10.3389/fphys.2020.00357

**Published:** 2020-04-28

**Authors:** Joanna F. Flatt, Christian J. Stevens-Hernandez, Nicola M. Cogan, Daniel J. Eggleston, Nicole M. Haines, Kate J. Heesom, Veronique Picard, Caroline Thomas, Lesley J. Bruce

**Affiliations:** ^1^Bristol Institute for Transfusion Sciences, National Health Service (NHS) Blood and Transplant, Bristol, United Kingdom; ^2^School of Biochemistry, University of Bristol, Bristol, United Kingdom; ^3^Assistance Publique-Hôpitaux de Paris, Service d’Hématologie Biologique, Hôpital Bicêtre, Paris, France; ^4^Faculté de Pharmacie, Université Paris-Saclay, Chatenay Malabry, France; ^5^Hématologie et Immunologie Pédiatrique, Hôpital Mère Enfants, Nantes, France

**Keywords:** Southeast Asian ovalocytosis, Band 3, SLC4A1, dyserythropoiesis, hereditary stomatocytosis, congenital dyserythropoiesis type II, cytokinesis, reticulocyte maturation

## Abstract

Southeast Asian Ovalocytosis results from a heterozygous deletion of 9 amino acids in the erythrocyte anion exchange protein AE1 (band 3). The report of the first successful birth of an individual homozygous for this mutation showed an association with severe dyserythropoietic anemia. Imaging of the proband’s erythrocytes revealed the presence of band 3 at their surface, a reduction in Wr(b) antigen expression, and increases in glycophorin C, CD44, and CD147 immunoreactivity. Immunoblotting of membranes from heterozygous Southeast Asian Ovalocytosis red cells showed a quantitative increase in CD44, CD147, and calreticulin suggesting a defect in reticulocyte maturation, as well as an increase in phosphorylation at residue Tyr359 of band 3, and peroxiredoxin-2 at the membrane, suggesting altered band 3 trafficking and oxidative stress, respectively. *In vitro* culture of homozygous and heterozygous Southeast Asian Ovalocytosis erythroid progenitor cells produced bi- and multi-nucleated cells. Enucleation was severely impaired in the homozygous cells and reduced in the heterozygous cells. Large internal vesicular accumulations of band 3 formed, which co-localized with other plasma membrane proteins and with the autophagosome marker, LC3, but not with ER, Golgi or recycling endosome markers. Immunoprecipitation of band 3 from erythroblast cell lysates at the orthochromatic stage showed increased interaction of the mutant band 3 with heat shock proteins, ubiquitin and cytoskeleton proteins, ankyrin, spectrin and actin. We also found that the mutant band 3 forms a strong interaction with non-muscle myosins IIA and IIB, while this interaction could not be detected in wild type erythroblasts. Consistent with this, the localization of non-muscle myosin IIA and actin was perturbed in some Southeast Asian Ovalocytosis erythroblasts. These findings provide new insights toward understanding *in vivo* dyserythropoiesis caused by the expression of mutant membrane proteins.

## Introduction

We previously reported on the only known case of homozygous Southeast Asian Ovalocytosis (SAO) (Picard et al., 2014), a condition caused by a mutation in *SLC4A1* the gene encoding erythrocyte anion exchanger 1 (AE1, band 3). The affected child was born prematurely with hydrops and severe anemia and developed distal renal tubular acidosis (dRTA) at 3 months. Bone marrow aspiration showed dyserythropoiesis. Unexpectedly, we found that some mature red blood cells (RBCs), containing SAO band 3 alone, were produced by the child’s bone marrow and survived in the circulation. These cells were very large, cigar-shaped and had an altered affinity for certain anti-band 3 antibodies. We were interested to further characterize these cells and examine the effect that the expression of SAO band 3 had on erythropoiesis.

SAO is caused by the heterozygous deletion of codons 400–408 in *SLC4A1*. The translated protein carries the corresponding deletion of 9 amino acid residues (Jarolim et al., 1991; Tanner et al., 1991). The deletion occurs at the boundary between the cytoplasmic N-terminal domain and the transport-active transmembrane domain. This mutation abolishes the anion exchange activity of band 3, and is associated with significant structural changes, nonetheless the mutant protein is successfully expressed at the plasma membrane (Tanner et al., 1991; Schofield et al., 1992). Studies have shown that SAO band 3 is able to interact with and modify normal band 3 expressed in the same cell (Kuma et al., 2002). Band 3 exists as dimers and tetramers in the red cell membrane, but SAO band 3 is believed to form higher oligomers and associate with the underlying erythrocyte cytoskeleton more readily than wild type band 3, offering an explanation for the observed increase in membrane rigidity in these cells (Mohandas et al., 1992; Sarabia et al., 1993; Liu et al., 1995).

SAO has a distinctive geographical distribution, with cases predominantly in Malaysia, The Philippines and Papua New Guinea, and has thus far only ever been described in the heterozygous state (Liu et al., 1994). This is despite a relatively high allele frequency of 5–20% in certain populations (Amato and Booth, 1977). SAO has been the subject of much interest, because the mutation confers some protection from cerebral malaria, a severe complication of *Plasmodium falciparum* infection (Allen et al., 1999) and also protects against infection by *Plasmodium* vivax (Rosanas-Urgell et al., 2012). It had previously been thought that *P. falciparum* cannot invade SAO cells as easily as control cells, but there is no clinical data to support this (Lin et al., 2010). Experiments have shown that reduced invasion by *P. falciparum* is at least partly explained by accelerated depletion of ATP levels in SAO cells *in vitro* (Dluzewski et al., 1992). The depletion of ATP is secondary to a cation leak caused by the mutant band 3 protein, and in this respect SAO is similar to another cation-leaky disorder, cryohydrocytosis (CHC; Guizouarn et al., 2011). The SAO mutation is unusual in the context of the currently known band 3 mutations producing a cation leak, since these are invariably point mutations leading to single amino acid substitutions occurring around the transport domain, transmembrane spans 9 and 10 (Bruce et al., 2005).

In the present study, we have studied blood and bone marrow samples from the affected child, homozygous for the SAO mutation, and blood samples from their heterozygous parents. Analysis of the mature red cells and of erythroid progenitor cells grown in culture revealed multiple changes in both homozygous and heterozygous SAO cells, including altered band 3 protein interactions and trafficking. Notably, the expression of SAO band 3 results in multinucleated erythroblasts, and reduced proliferation and enucleation, producing a dyserythropoietic phenotype.

## Materials and Methods

### Patients

The homozygous SAO patient and heterozygous SAO parents have been described (Picard et al., 2014). In brief, the child was born prematurely with hydrops, and severe anemia which was treated with monthly transfusions. Distal renal tubular acidosis (dRTA) developed at 3 months and was treated with sodium bicarbonate and potassium gluconate. The severe anemia was caused in part by hemolysis (perhaps aggravated by the co-inherited hemoglobin defects) but also from deficient red cell production. Genetic analyses indicated that the child inherited a heterozygous 3.7 kb alpha-globin deletion (HBA1 HBA2-3.7 kb del) from the mother, a heterozygous beta-globin variant “La Desirade” (resulting from HBB c.389 C > T) from the father, and homozygous SAO. He did not inherit his mother’s sickle cell trait (HBB c.20 C > T). Hb “La Desirade” is asymptomatic in heterozygotes and could not account for the early severe anemia because beta-globin is not significantly expressed at 22 weeks gestation. The heterozygous -3.7 alpha thalassemia trait has usually very mild consequences on erythropoiesis and is totally asymptomatic, except for minor microcytosis, even when homozygous. Indeed, the mother has a homozygous -3.7 alpha thalassemia trait (plus a beta globin S variant) with no anemia. Bone marrow aspirate from the child showed binuclearity, karyorrhexis and macrocytosis, features typical of dyserythropoiesis. The child is now 10 years old and quite well, with regular transfusions, iron chelation and acidosis treatment. Written informed consent was obtained from both parents for themselves and the child. This study is part of a larger study approved by the National Health Service National Research Ethics Service South West entitled “*In Vitro* Studies of Erythropoiesis in Health and Disease.”

### Erythrocyte Membrane Protein Analysis

Preparation of erythrocyte membranes, SDS-PAGE and Western blotting analysis of heterozygous SAO membranes were carried out as previously described (Bruce et al., 2003). Antibodies are listed in Table 1. Blots were analyzed using semi-quantitative scanning densitometry with the use of ImageJ software^1^ and ImageStudio (LI-COR Biosciences).

**TABLE 1:** Antibodies used in the study.

**Protein/antigen**	**Antibody/clone**	**Company**	**Product no.**	**Species**	**Technique**
Band 3	BRIC170	IBGRL		Mouse	IF/WB
Band 3	BRIC155	IBGRL		Mouse	IF
Band 3	BRIC132	IBGRL		Mouse	IF
Band 3	BRAC17	IBGRL		Rat	IF
Band 3	BRAC66	IBGRL		Rat	IP
Phospho-B3 Y8	polyclonal	In-house		Rabbit	WB
Phospho-B3 Y359	polyclonal	In-house		Rabbit	IF/WB
Phospho- B3 Y904	polyclonal	In-house		Rabbit	WB
Protein 4.2	polyclonal	In-house		Rabbit	WB
GPA	BRIC256	IBGRL		Mouse	IF
GPA	polyclonal	In-house		Rabbit	IF/WB
Wr(b)	BIRMA84b	IBGRL		Mouse	IF
Wr(b)	BRIC13	IBGRL		Mouse	IF
Wr(b)	BRAC13	IBGRL		Rat	IF
GPB (and to a lesser extent GPA)	R1.3	IBGRL		Mouse	IF/WB
RhAG	LA1818	IBGRL		Mouse	IF
RhAG	polyclonal	In-house		Rabbit	WB
Rh	polyclonal	In-house		Rabbit	WB
Rh	BRIC69	IBGRL		Mouse	IF
CD47	BRIC211	IBGRL		Mouse	IF
CD47	polyclonal	In-house		Rabbit	WB
ICAM-4	BS46/BS56	IBGRL		Mouse	WB
GPC	BRIC10	IBGRL		Mouse	IF
GPC	polyclonal	In-house		Rabbit	IF/WB
CD44	BRIC235	IBGRL		Mouse	IF/WB
CD44	polyclonal	In-house		Rabbit	IF
Ankyrin	BRIC274	IBGRL		Mouse	IF
Alpha-spectrin	BRIC174	IBGRL		Mouse	IF/WB
Beta-spectrin	BRAC65	IBGRL		Rat	WB
NMMIIA	polyclonal	Abcam	ab24762	Rabbit	IF/WB
NMMIIB	polyclonal	Abcam	ab24761	Rabbit	WB
Glut1	polyclonal	Gift from S. Baldwin		Rabbit	IF
Stomatin	GARP50	Gift from R. Prohaska		Mouse	IF
Stomatin	polyclonal	In-house		Rabbit	IF/WB
SLP-2	polyclonal	In-house		Rabbit	WB
Aquaporin-1	polyclonal	In-house		Rabbit	IF/WB
CD147	MEM-M6/1	AbD Serotec	MCA1876	Mouse	IF
CD147	EPR4053	Abcam	ab108308	Rabbit	WB
Lutheran	BRIC221	IBGRL		Mouse	WB
LFA-3 (CD58)	BRIC5	IBGRL		Mouse	WB
DAF (CD55)	BRIC128	IBGRL		Mouse	WB
Transferrin receptor	polyclonal	Abcam	ab84036	Rabbit	IF/WB
Calreticulin	polyclonal	Abcam	ab2907	Rabbit	IF/WB
Giantin	polyclonal	Covance	PRB-114C	Rabbit	IF
LAMP1	polyclonal	Abcam	ab24170	Rabbit	IF
LAMP2	monoclonal	Abcam	ab25631	Mouse	WB
LC3	polyclonal	MBL	PM036	Rabbit	IF
Pericentrin	polyclonal	Abcam	ab4448	Rabbit	IF
CD63	H5C6	BD Biosciences	556019	Mouse	IF
VDAC1	polyclonal	Abcam	ab15895	Rabbit	IF/WB
Peroxiredoxin 2	EPR5154	Abcam	ab109367	Rabbit	WB
Beta-actin	AC-15	Abcam	ab6276	Mouse	WB

*IF, immunofluorescence; IP, immunoprecipitation; WB, Western blotting.*

### Culture of CD34^+^ Cells

CD34^+^ cells were isolated from a 3 ml sample of bone marrow from the proband and 50 ml peripheral blood samples from the parents and controls by positive selection using paramagnetic microbeads (Miltenyi Biotec, Germany) according to the manufacturer’s instructions and cultured as described (Flatt et al., 2011). Briefly, cells were cultured at 37°C in a humidified atmosphere of 5% CO_2_ in air. The base culture medium used throughout comprised IMDM (Biochrom, Germany) supplemented with 3% AB serum, 10 μg/ml insulin, 3 IU/ml heparin (all from Sigma-Aldrich, Poole, United Kingdom), 2% FCS (Hyclone, Fisher Scientific, Ltd., United Kingdom) 3 U/ml EPO (Roche, Basel, Switzerland) and 200 μg/ml holotransferrin (R&D Systems Minneapolis, MN, United States). On days 0–8 cells were cultured in base medium with 40 ng/ml SCF and 1 ng/ml IL3 (R&D systems). From days 9 to 12, in base medium with 10 ng/ml SCF and from day 13 onward in base medium with an increased holotransferrin concentration of 500 μg/ml. Cytomicrographs were prepared by cytocentrifugation (Shandon) of 2–4 × 10^4^ cells onto slides at 1350 rpm for 5 min and stained with Leishman’s (VWR) according to the manufacturer’s instructions.

### Confocal Microscopy

Peripheral blood samples from the homozygous SAO proband were obtained immediately before transfusion. Confocal imaging was carried out as described (Flatt et al., 2011). Briefly, erythrocytes (5 × 10^5^ cells/slide) were fixed using 1% formaldehyde, 0.0075% glutaraldehyde and permeabilized with 0.1% Triton-X100. Reticulocytes and late stage erythroblasts (5 × 10^5^ cells/slide) were fixed using 1% formaldehyde and permeabilized using 0.01% saponin. Early stage erythroblasts (5 × 10^5^ cells/slide) were fixed using 3% formaldehyde and permeabilized with 0.01% digitonin. Antibodies are listed in Table 1.

### Cultured Cell Lysate Analysis

At selected time points 2 × 10^6^ cells were removed from the culture, washed once in PBS, then solubilized in 200 μl erythroblast lysis buffer (20 mM Tris-HCl, pH 7.5, 10% glycerol, 150 mM NaCl, 1% Triton-X100, 0.1% SDS, 2 mM PMSF, 1× protease inhibitor cocktail). Protein concentration was estimated using Bradford’s reagent. Lysates were separated by SDS-PAGE on a 10% acrylamide gel. Equal amounts of protein (usually 10 μg) were loaded per gel track. Gels were immunoblotted as described (Bruce et al., 2003).

### Immunoprecipitation of Proteins

Cultured homozygous SAO cells (4 × 10^6^) at day 27, and cultured control cells (4 × 10^6^) at day 14 matching the stage of the SAO cells as closely as possible (predominantly orthochromatic erythroblasts, ∼10% enucleated) were solubilized in NP40-IP buffer (1% Non-idet-P40, 150 mM KCl, 10 mM Tris-HCl, pH 7.4) and immunoprecipitated using protein G sepharose beads preloaded with rat monoclonal anti-band 3 (BRAC66) as described (Bruce et al., 2003). Immunoprecipitated proteins, using anti-band 3 (BRAC66) were separated by SDS-PAGE and analyzed by LC-MS/MS.

### Protein Identification for Immunoprecipitates

Immunoprecipitated proteins, using anti-band 3 (BRAC66) antibody, were separated by SDS-PAGE. The gel lane was cut into 3 slices and each slice subjected to in-gel tryptic digestion using a ProGest automated digestion unit (Digilab United Kingdom). The resulting peptides were fractionated using a Dionex Ultimate 3000 nanoHPLC system in line with an LTQ-Orbitrap Velos mass spectrometer (Thermo Fisher Scientific). In brief, peptides in 1% (vol/vol) formic acid were injected onto an Acclaim PepMap C18 nano-trap column (Dionex). After washing with 0.5% (vol/vol) acetonitrile 0.1% (vol/vol) formic acid peptides were resolved on a 250 mm × 75 μm Acclaim PepMap C18 reverse phase analytical column (Dionex) over a 150 min organic gradient, using 7 gradient segments (1–6% solvent B over 1 min, 6–15% B over 58 min., 15–32%B over 58 min, 32–40%B over 3 min, 40–90%B over 1 min., held at 90%B for 6 min and then reduced to 1%B over 1 min.) with a flow rate of 300 nl min^–1^. Solvent A was 0.1% formic acid and Solvent B was aqueous 80% acetonitrile in 0.1% formic acid. Peptides were ionized by nano-electrospray ionization at 2.1 kV using a stainless steel emitter with an internal diameter of 30 μm (Thermo Fisher Scientific) and a capillary temperature of 250°C. Tandem mass spectra were acquired using an LTQ- Orbitrap Velos mass spectrometer controlled by Xcalibur 2.1 software (Thermo Fisher Scientific) and operated in data-dependent acquisition mode. The Orbitrap was set to analyze the survey scans at 60,000 resolution (at m/z 400) in the mass range m/z 300–2000 and the top twenty multiply charged ions in each duty cycle selected for MS/MS in the LTQ linear ion trap. Charge state filtering, where unassigned precursor ions were not selected for fragmentation, and dynamic exclusion (repeat count, 1; repeat duration, 30 s; exclusion list size, 500) were used. Fragmentation conditions in the LTQ were as follows: normalized collision energy, 40%; activation q, 0.25; activation time 10 ms; and minimum ion selection intensity, 500 counts.

The raw data files were processed and quantified using Proteome Discoverer software v1.2 (Thermo Fisher Scientific) and searched against the SwissProt Human database (122604 entries) using the SEQUEST algorithm. Peptide precursor mass tolerance was set at 10 ppm, and MS/MS tolerance was set at 0.8 Da. Search criteria included carbamidomethylation of cysteine (+57.0214) as a fixed modification and oxidation of methionine (+15.9949) as a variable modification. Searches were performed with full tryptic digestion and a maximum of 1 missed cleavage was allowed. The reverse database search option was enabled and all peptide data was filtered to satisfy false discovery rate (FDR) of 5%.

## Results

### Homozygous SAO Erythrocytes Are Large and Aberrantly-Shaped With Altered Membrane Protein Expression

**FIGURE 1:**
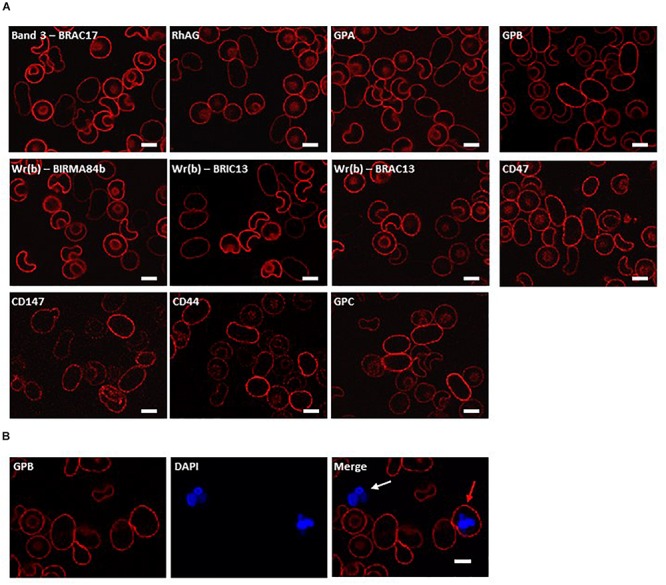
Immunocytochemistry of homozygous SAO erythrocytes. Circulating erythrocytes from the homozygous SAO individual, containing both large, aberrantly-shaped homozygous SAO cells and normal biconcave donor cells, were imaged using immunofluorescent labeling and confocal microscopy. **(A)** Red cells were reacted with monoclonal antibodies directed to extracellular epitopes of membrane proteins of interest: Band 3 (BRAC17), RhAG (LA1818), GPA (BRIC256), GPB (and to a lesser extent GPA) (R1.3), Wr(b) (BIRMA 84b, BRIC13 and BRAC13), CD47 (BRIC211), CD147 (MEM6-6), CD44 (BRIC235), and GPC (BRIC10). **(B)** Red cells were co-stained with erythroid marker anti-GPB (and to a lesser extent GPA, R1.3; red) and DNA dye DAPI (blue). The white arrow indicates a leukocyte, the red arrow indicates an erythroid cell that has retained nuclear material. Numbers of nucleated erythroid cells were not sufficient to quantify accurately but were seen occasionally in the confocal slides of homozygous SAO peripheral blood whereas they are very rare in control slides. Images are representative of at least 5 different fields from one experiment. Scale bars represent 5 μm.

In the clinical report of this patient we showed that homozygous SAO cells were clearly identifiable amongst the normal transfused donor red cells based on morphology and reduced reactivity with anti-band 3 monoclonal antibody BRIC6 (Smythe et al., 1995). The homozygous SAO cells can be very large (11–13 μm in length) and have grossly abnormal morphology. Cells are predominantly cigar-shaped, and some show a central cleft (Figure 1). These morphological changes probably result in cytoskeletal stretching, and affect protein packing, modifying the accessibility and presentation of epitopes. Indeed, apparent altered antigen expression in heterozygous SAO cells has been reported (Booth et al., 1977).

In order to investigate further changes in the SAO membrane, intact cells were incubated with various antibodies to extracellular epitopes of erythrocyte membrane proteins. The band 3 monoclonal antibody BRAC17 showed reduced binding compared to the donor cells (Figure 1A), as reported (Smythe et al., 1995; Picard et al., 2014). Similarly, anti-Rh-associated glycoprotein (RhAG) and anti-glycophorin A (GPA) showed slightly reduced immunoreactivity (Figure 1A), and reduced reactivity occurred with antibodies directed against the Wr(b) antigen which is formed by an interaction between GPA and band 3 (Bruce et al., 1995). Anti-glycophorin B (GPB) and anti-CD47 showed similar staining between the SAO and donor RBCs (Figure 1A). Anti-CD147, anti-CD44 and anti-glycophorin C (GPC) showed increased staining (Figure 1A). Increased CD147 staining was of interest because this protein has been identified as a universal receptor for *P. falciparum* invasion of red cells (Crosnier et al., 2011).

Nucleated erythroid cells were also detected in the homozygous SAO circulating red cell population, indicating their premature egress from the bone marrow (Figure 1B).

**FIGURE 2:**
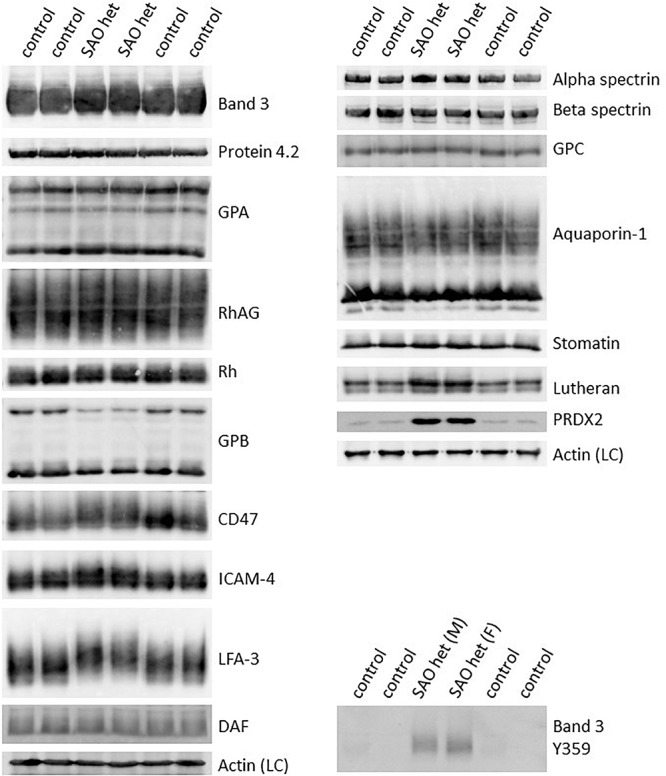
Altered expression of membrane proteins in heterozygous SAO red cells. Red cell membranes from SAO and control peripheral blood samples were prepared, separated by SDS-PAGE and immunoblotted. The antibodies directed against protein 4.2, GPA, RhAG, Rh polypeptides, CD47, aquaporin-1, GPC, stomatin and phospho-Tyr359 band 3 were in-house rabbit polyclonal antibodies. Commercial antibodies were used to detect actin (ab6276) and peroxiredoxin-2 (PRDX2; ab109367) both from Abcam, Cambridge, United Kingdom. Monoclonal antibodies were used to detect band 3 (BRIC170), alpha-spectrin (BRIC174), beta-spectrin (BRAC65), GPB (and to a lesser extent GPA, R1.3), ICAM-4 (BS46/BS56), LFA-3 (BRIC5), DAF (BRIC128) and Lutheran (BRIC221). SAO het and SAO het (F): Membranes prepared from the heterozygous father of the homozygous SAO child. SAO het (M): Membranes prepared from the heterozygous mother of the homozygous SAO child. LC, Loading control.

These changes in antibody staining in the confocal imaging analysis of SAO RBCs may reflect a quantitative change in protein expression, or a change in epitope presentation. Homozygous SAO RBC membranes were not available (as the patient was regularly transfused) so heterozygous SAO RBC membranes were analyzed by immunoblotting. Relative to control membranes, band 3, protein 4.2, GPA, RhAG, CD47 and Rh proteins were present in normal amounts, whereas GPB and aquaporin 1 (AQP1) were slightly reduced (Figure 2). Heterozygous SAO RBC membranes showed a 3-fold increase in the amount of Lutheran protein by immunoblotting but no difference in glycophorin C (GPC) (Figure 2). Strikingly, the membrane-associated anti-oxidant enzyme peroxiredoxin-2 (PRDX2) was 20-fold increased (Figure 2).

**FIGURE 3:**
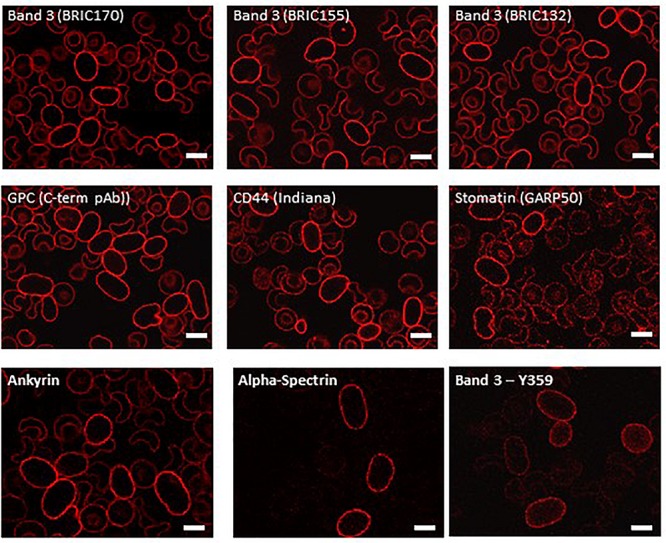
Immunocytochemistry of permeabilized homozygous SAO erythrocytes. Circulating erythrocytes from the homozygous SAO individual were fixed, permeabilized and imaged using immunofluorescent labeling and confocal microscopy. Antibodies directed against intracellular epitopes of plasma membrane proteins. Mouse monoclonal antibodies were used to detect band 3 (BRIC170, BRIC155, BRIC132), stomatin (GARP50), ankyrin (BRIC274) and alpha-spectrin (BRIC174). In-house rabbit polyclonal antibodies were used to detect GPC, CD44 and phospho-Tyr359 band 3. Scale bars represent 5 μm.

Examination of intracellular epitopes using α-spectrin and ankyrin antibodies and confocal imaging showed increased staining in homozygous SAO cells (Figure 3), but no quantitative increase was seen in α-spectrin in the heterozygous SAO immunoblots (Figure 2). Similarly, staining with intracellular band 3 antibodies (BRIC170, BRIC155, BRIC132) and intracellular antibodies to GPC and stomatin showed increased staining in homozygous SAO cells (Figure 3), but no quantitative increase was seen in the corresponding heterozygous SAO immunoblots (Figure 2), suggesting that the increased staining in the confocal imaging analysis of many of the intracellular epitopes may be due to an increased permeability of the SAO membrane.

In contrast, staining produced by an antibody specific to tyrosine phosphorylation of band 3 at residue 359 was increased in SAO homozygote erythrocytes (Figure 3) and immunoblotting confirmed the increased band 3 tyrosine phosphorylation at residue 359 in heterozygous SAO membranes (Figure 2). Phosphorylation at tyrosine residue 904 was absent in both SAO and control cells (data not shown).

Of note, a number of proteins migrated more slowly than wild type in SDS-PAGE, consistent with hyperglycosylation and suggestive of altered trafficking (Figure 2). These proteins included band 3, CD47, ICAM-4, and LFA-3, some of which are known to associate with band 3 in RBC membrane complexes (Bruce et al., 2003) and may be trafficked together with band 3 in the internal membranes.

### Cultured SAO Cells Show a Dyserythropoietic Phenotype

**FIGURE 4:**
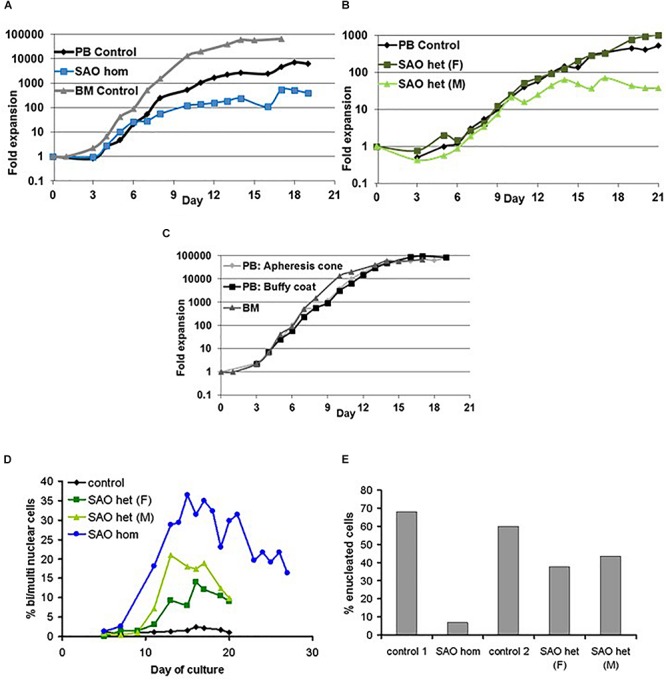
Proliferation of cultured CD34^+^ cells. Control, heterozygous SAO and homozygous SAO CD34^+^ cells were cultured, and their fold-increase in cell number is shown, plotted against the day of culture. **(A)** Expansion of homozygous SAO (blue line) and control (black line) CD34^+^ cells in culture. The SAO CD34^+^ cells were isolated from 3 ml of bone marrow (BM), the control CD34^+^ cells from peripheral blood (PB; 50 ml). A further control CD34^+^ culture grown from frozen BM CD34^+^ cells, but at a different time and therefore not directly comparable, is shown (gray line). **(B)** Expansion of heterozygous SAO (SAO [father (F) dark green line]; SAO [mother (M) pale green line]) and control (black line) CD34^+^ cells in culture. All three cultures were grown from CD34^+^ cells isolated from peripheral blood (PB; 50 ml). **(C)** Comparison of the typical rate of expansion of three controls where the CD34^+^ cells were isolated from different sources. Expansion of adult derived CD34^+^ cells in culture from a fresh apheresis cone (pale gray line), fresh buffy coat (black line) or from frozen bone marrow (gray line). **(D)** Graph showing the percentage of cells with two or more nuclei over the course of the cultures. Cells were counted from cytomicrographs, and counts were averaged from 3 different fields. Numbers of cells counted per field were 125 ± 48 (s.d.). **(E)** Graph showing the percentage of cells that had successfully enucleated at day 20 in culture. Cells were counted from cytomicrographs, and counts were averaged from 5 different fields. Numbers of cells counted per field were 136 ± 45 (s.d.).

**FIGURE 5:**
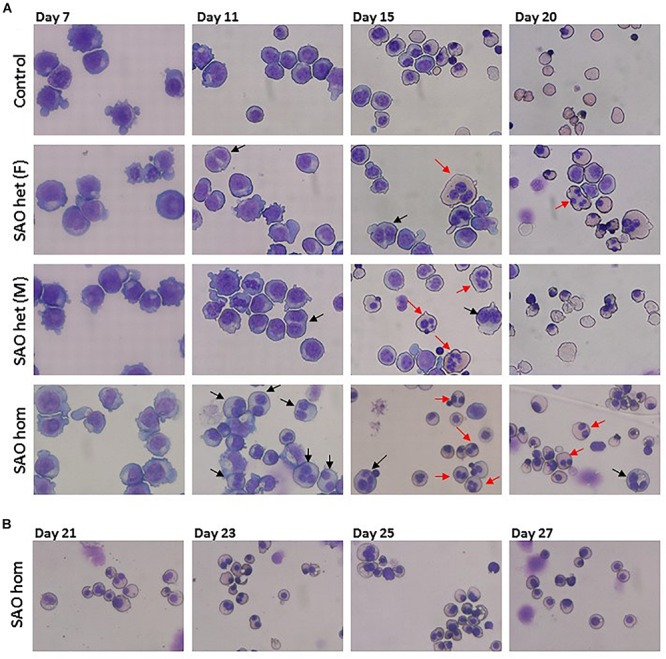
Cytomicrographs of cultured control and SAO CD34^+^ cells. **(A)** Comparison of control with heterozygous SAO [(F) = father, (M) = mother] and homozygous SAO cells over 20 days of culture. Bi-nuclear proerythroblasts and basophilic erythroblasts are indicated with black arrows. Multi-nuclear polychromatophilic and orthochromatic erythroblasts are indicated with red arrows. **(B)** Homozygous SAO cells cultured for a further 7 days did not achieve a significant level of enucleation.

CD34^+^ cells from the bone marrow of the homozygous SAO proband were cultured alongside control CD34^+^ cells from peripheral blood of a healthy volunteer. Control cells showed normal proliferation and differentiation (Figures 4A–C). In contrast, proliferation of homozygous SAO cells was normal at early stages of the culture, but reduced once band 3 started to be expressed around day 7 (Figure 4A). This reduction in the rate of proliferation of homozygous SAO cells had a number of causes but cannot be attributed to a difference in stem cell source (Figure 4C). Numerous dead cells and debris were noted in the homozygous SAO culture post day 11. Homozygous SAO cells also exhibited high numbers of multinucleated cells, coinciding with the time that band 3 is expressed (Figure 4D). The multiple nuclei in these cells suggest a defect in cytokinesis, the final stage of cell division. Although in the later stages of the culture, the percentage of multinucleated cells was observed to decrease slightly (Figures 4D, 5). In later stages of the culture the low cell numbers may have been caused in part by the instability of the very large reticulocytes formed. It was also noted that the homozygous SAO cells failed to enucleate efficiently, reaching only 7% enucleation after 20 days in culture compared to 68% in controls (Figure 4E).

In a separate experiment CD34^+^ cells from the peripheral blood of the heterozygous SAO parents of the proband were cultured alongside control CD34^+^ cells from peripheral blood of a healthy volunteer. The heterozygous SAO cells from the father showed normal proliferation, whereas the cells from the mother grew less well (Figure 4B). Unexpectedly, the heterozygous SAO erythroblasts from both parents also displayed multinuclearity, although to a lesser extent (Figures 4D, 5). Heterozygous SAO cells achieved 40–45% enucleation, compared to 60% in the control, at day 20 (Figure 4E).

### Internal Vesicles of SAO Band 3 Co-stain With Other Membrane Proteins

**FIGURE 6:**
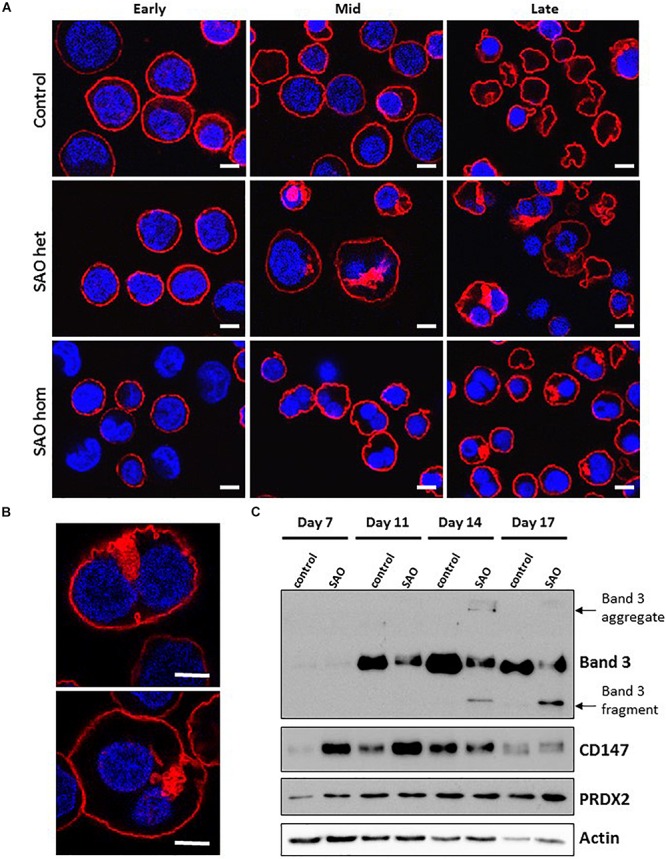
Expression of SAO band 3 throughout erythropoiesis. Control, heterozygous (father of proband) and homozygous SAO CD34^+^ cells were cultured for 21 days and their band 3 expression was monitored by the use of monoclonal anti-band 3 antibody after fixation and permeabilization. Confocal microscope images are shown from 3 different time-points: Early (day 10), Mid (day 14) and Late (day 21). **(A)** Comparison of control, heterozygous and homozygous SAO cells using BRAC17 (red) and DAPI (blue). Early homozygous SAO erythroblasts express very little band 3 probably because SAO band 3 is mis-folded and has problems trafficking to the red cell membrane. The difference in size and morphology of cells, especially notable in heterozygous SAO cells mid-culture is due to the formation of the large aberrantly-shaped SAO cells that eventually become SAO red cells. Scale bars represent 5 μm. **(B)** Higher magnification images of day 17 heterozygous SAO cells exhibiting internal clusters of band 3 vesicles. Staining shows Band 3 (BRAC17; red) and nuclei (DAPI; blue). Images are representative of at least 5 different fields from one experiment. Scale bars represent 5 μm. **(C)** Immunoblot of cultured homozygous SAO and control cell lysates probed with anti-band 3 (BRIC170); anti-CD147 (rabbit monoclonal antibody; ab108308); anti-PRDX2 (ab109367) and loading control anti-actin (ab6276).

In both heterozygous and homozygous RBCs SAO band 3 was successfully expressed at the erythroblast cell membrane throughout erythropoiesis (Figure 6A). However, at later stages of erythropoiesis it was present in large areas of dense staining in some SAO erythroblasts, but not in controls (Figure 6A). These were often located in the region between nuclei in bi-nuclear cells, and across the midbody area where the cytokinesis furrow is expected to form. Higher magnification imaging of heterozygous SAO cells revealed that these areas are composed of numerous clustered band 3-positive vesicles (Figure 6B). Immunoblots of cultured cell lysates showed reduced levels of band 3 and the presence of higher and lower molecular weight bands that probably represent aggregated band 3 and proteolytic fragments in homozygous SAO respectively. Again, it was noted that SAO band 3 migrated more slowly than wild type in SDS-PAGE, consistent with hyperglycosylation and suggestive of altered trafficking (Figure 6C). CD147 levels were found to be increased in homozygous SAO lysates relative to control in the early stages of culture (days 7 and 11) and total PRDX2 levels only began to increase relative to control in late culture (day 17).

**FIGURE 7:**
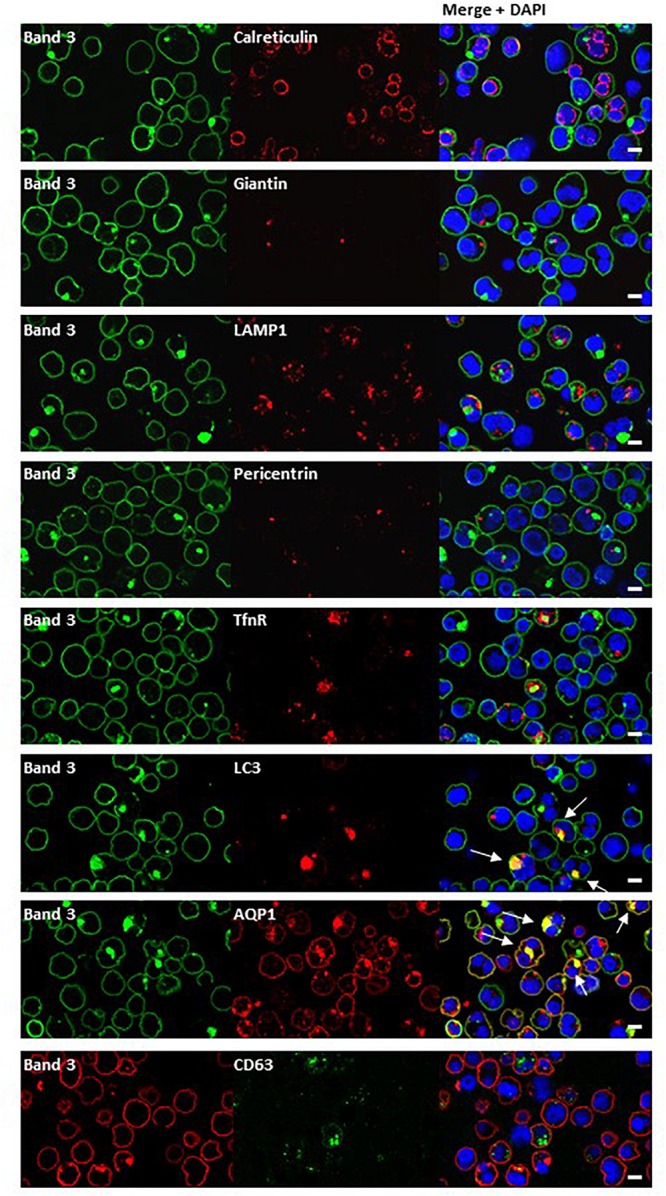
Immunocytochemistry of homozygous SAO erythroblasts. Late stage homozygous SAO cells were dual stained using antibodies against band 3 (BRIC170, green; or BRAC17, red) and markers for plasma membrane, internal compartments or organelles. Markers used were: Calreticulin (endoplasmic reticulum; ab2907), Giantin [Golgi; PRB-114C (Covance)], LAMP1 (lysosome associated membrane protein 1; lysosome; ab24170), Pericentrin (microtubule organizing center; ab4448), TfnR (Transferrin receptor/CD71; ab84036), LC3 [microtubule-associated proteins 1A/1B light chain 3A; autophagosome; PM036 (MBL)], AQP1 (Aquaporin-1; plasma membrane), and CD63 [recycling endosome; 556019 (BD Biosciences)]. Colocalization is indicated with white arrows (yellow areas in merged images). Images are representative of at least 5 different fields from one experiment. Scale bars represent 5 μm.

**FIGURE 8:**
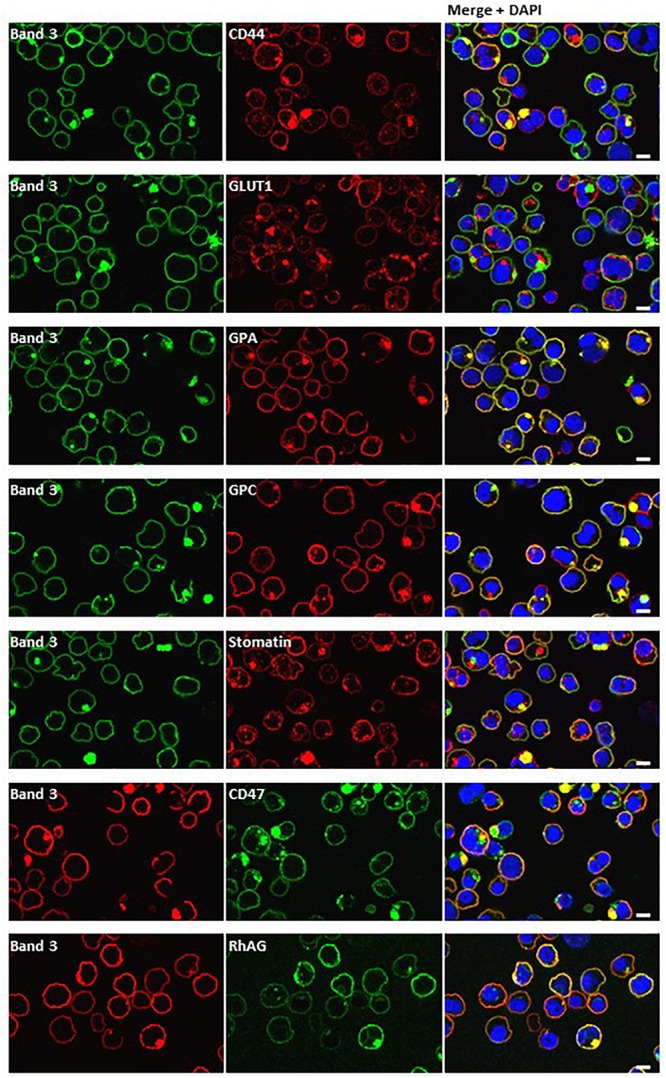
Dual staining with plasma membrane markers. Late stage homozygous SAO cells were fixed, permeabilized and dual stained with anti-band 3 (BRIC170, green; or BRAC17, red) and antibodies against plasma membrane proteins. Antibodies used were rabbit polyclonals against CD44, GLUT1 (gift from Prof. S. Baldwin), GPA, GPC and mouse monoclonals against stomatin (gift from Prof. R. Prohaska; GARP50), CD47 (BRIC211) and RhAG (LA1818). Yellow areas in merged images indicate colocalization. Scale bars represent 5 μm.

Dual staining of band 3 with compartmental markers showed strong colocalization with the autophagosome marker LC3, but not with secretory pathway markers (Figure 7). Aquaporin-1 acted as a marker for the plasma membrane and showed strong colocalization with the band 3 aggregates (Figure 7). Other membrane proteins (CD44, GPA, GPC, stomatin, CD47, RhAG) also showed positive staining in the areas of aggregated band 3 (Figure 8), although the glucose transporter 1 (Glut1) formed aggregates that did not appear to colocalize with aggregated band 3. Membrane proteins that colocalized included proteins not known to associate directly with band 3 (CD44, GPC) suggesting that the origin of some of these vesicles may be endocytosis from the plasma membrane, although it is more probable that the vesicles arise from a problem in trafficking of membrane from Golgi to surface.

### Analysis of Proteins Associated With Band 3

**FIGURE 9:**
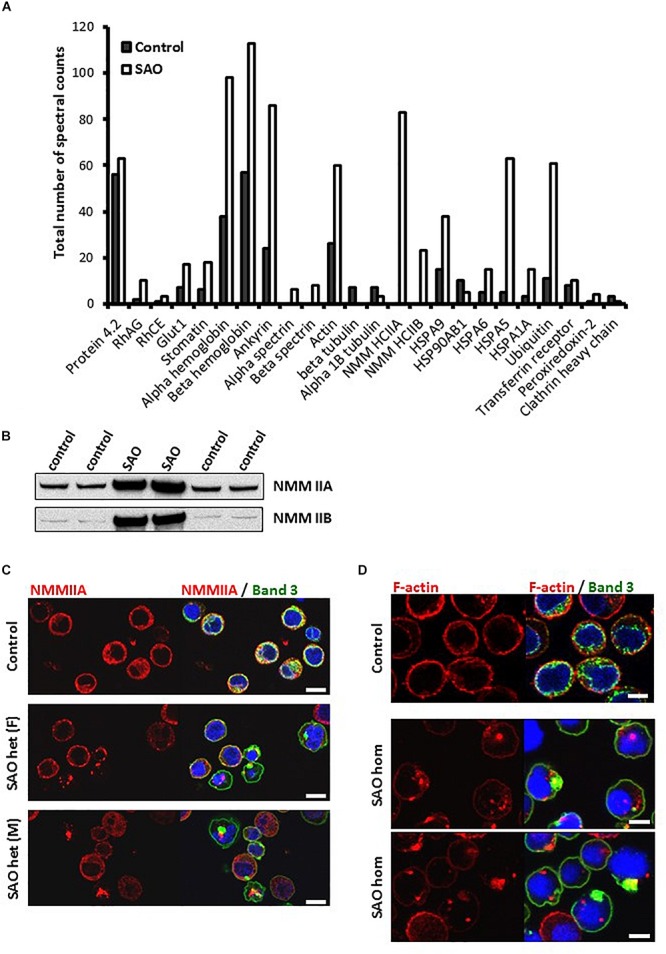
SAO band 3 immunoprecipitation. **(A)** Band 3 was immunoprecipitated from control (day 14) and homozygous SAO (day 27) cultured CD34^+^ cell lysates. These stages were not ideal but were chosen in order to match both cell types as closely as possible (predominantly orthochromic erythroblasts and 10% enucleated as shown in Figure 4). The immunoprecipitate was analyzed by mass spectrometry. The number of spectral counts obtained for the co-immunoprecipitated proteins from each culture are shown. **(B)** Immunoblots of control and heterozygous SAO mature erythrocyte membranes. Antibodies used were anti-NMMIIA (ab24762) and anti-NMMIIB (ab24761). **(C)** Day 14 control and heterozygous SAO cells were stained with anti-NMMIIA antibody (red) and anti-band 3 antibody (BRIC170; green). DNA was stained with DAPI (blue). Scale bars represent 10 μm. **(D)** Day 14 cells imaged using anti-band 3 antibody (BRIC170; green) and AlexaFluor 546-conjugated phalloidin, which binds F-actin (red). DNA was stained with DAPI (blue). Images are representative of at least 5 different fields from one experiment. Scale bars represent 5 μm.

To identify which proteins were associating with band 3, band 3 immunoprecipitates (IP) from lysates of cultured homozygous SAO cells and control cells that were as closely stage-matched as possible were analyzed by Mass Spectrometry. The spectral count obtained for certain proteins were compared between control and SAO (Figure 9A). Band 3 appeared more strongly in the control [1239 peptide spectral matches (PSMs) vs. 573 PSMs in SAO]. In contrast, many of the known binding partners of band 3 showed an increase in the SAO IP, including RhAG and GLUT1 (Bruce et al., 2003). Of interest, SAO band 3 pulled down four times as much alpha and beta globin than did WT band 3 (Figure 9A). More PSMs were also identified for ankyrin, spectrin and actin, consistent with an increased association of SAO band 3 with cytoskeletal proteins (Mohandas et al., 1992; Sarabia et al., 1993; Liu et al., 1995).

Significant levels of non-muscle myosin proteins IIA and IIB were identified in the SAO IP but none in the control (Figure 9A). Heterozygous SAO red cell membranes contained an increased amount of both proteins (Figure 9B). Non-muscle myosin II (NMMII) is involved in contractile ring formation in cytokinesis, and non-muscle myosin IIB plays a crucial role in erythroblast enucleation (Ubukawa et al., 2012; Moura et al., 2018). It is possible that in healthy erythroid cells band 3 and NMMII do interact during cell division, but that the transient nature of the interaction is not detectable by co-immunoprecipitation. SAO band 3 may form stronger interactions with NMMII, as it does with other cytoskeletal components, and disrupt the normal localization and function of myosin during the final stage of cell division. This hypothesis was examined by immunofluorescence of cultured control and heterozygous SAO cells. In normal nucleated cells NMMIIA shows a diffuse cytoplasmic staining pattern, which later also localizes at the plasma membrane (Figure 9C). NMMIIA staining was condensed and punctate in some of the nucleated heterozygous SAO erythroblasts containing band 3 aggregates (Figure 9C). Although colocalization between the two proteins was not observed, they frequently appeared immediately adjacent to each other (Figure 9C). Similarly, the cytoskeletal protein actin is important for both cytokinesis and enucleation (Konstantinidis et al., 2012). The increased association with band 3 implied by our proteomic data and the dyserythropoietic phenotype prompted us to study its localization in late stage homozygous SAO erythroblasts. In healthy cells F-actin staining is restricted to the periphery of the cell, at the plasma membrane (Figure 9D). However, in some SAO cells the staining pattern was internal and punctate, often located within regions of aggregated band 3 staining (Figure 9D).

The proteomics data also suggested that more Heat Shock 70 Proteins (HSP70) are associated with band 3 in homozygous SAO cells, in particular HSP70-5 (HSPA5; also known as GRP78 and/or BiP). In healthy cells, when misfolded proteins are produced, they are initially dealt with by these chaperone proteins that aid in the correct folding or refolding of the protein in the endoplasmic reticulum. HSP70-5 plays an important role in regulating the unfolded protein response (Brodsky and Skach, 2011). If chaperone-mediated refolding is unsuccessful the cell destroys the misfolded protein, primarily via ubiquitination and proteasomal degradation. Our data showed a 6-fold increase in amounts of ubiquitin associated with SAO band 3, or one of the other co-immunoprecipitated proteins within the SAO band 3 IP (Figure 9A), consistent with its targeting for degradation by the proteasome. Ubiquitination is also implicated in tagging proteins for internalization from the plasma membrane (Barriere et al., 2006).

### Sorting of Membrane Proteins at Enucleation

**FIGURE 10:**
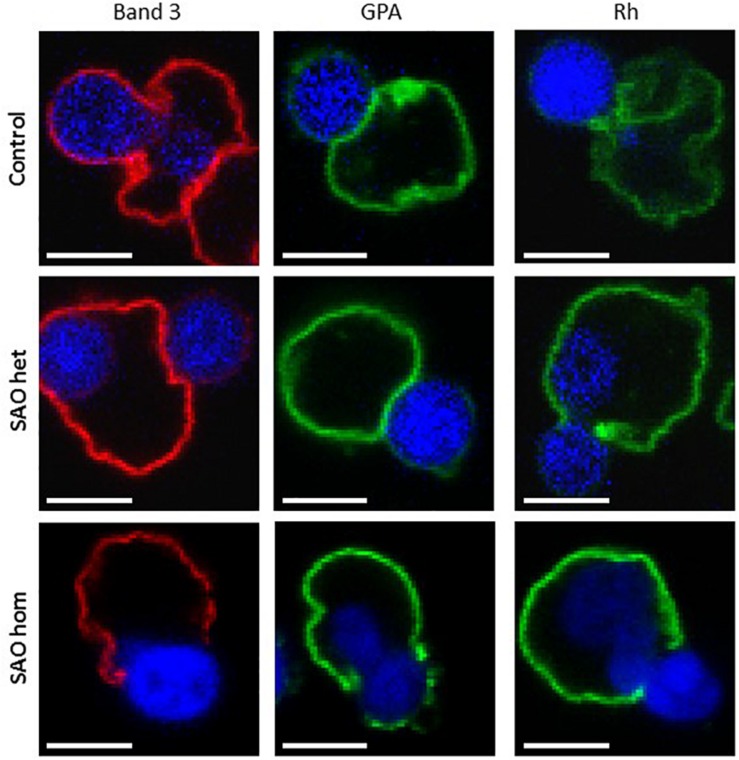
Immunocytochemistry of enucleating cultured CD34^+^ cells. Late stage control, heterozygous SAO and homozygous SAO cells were fixed, permeabilized and imaged using antibodies directed against band 3 (BRAC17), GPA (BRIC256) and Rh (BRIC69). Images are representative of at least 23 (band 3), 10 (GPA) or 5 (Rh) similar cells observed. Scale bars represent 5 μm.

Mutations in membrane proteins can give rise to mis-sorting of partner proteins during enucleation (Salomao et al., 2010; Satchwell et al., 2015). We used immunofluorescence to determine whether this occurs in cells expressing SAO band 3. In control cells band 3 was seen around both the reticulocyte and the extruding nucleus. This is consistent with previous observations using human cells (Lee et al., 2004; Bell et al., 2013). In contrast, very little was seen around the extruding nucleus in heterozygous SAO cells and none whatsoever in homozygous SAO cells (Figure 10). This suggests that all the band 3 in these cells is cytoskeletally-attached, as it is entirely restricted to the nascent reticulocyte.

Rh polypeptide sorting was unaffected in SAO cells, however homozygous SAO cells showed increased GPA staining around the nucleus compared to heterozygote and control cells (Figure 10). This is consistent with a weakened band 3-GPA interaction, as suggested by the depression in Wr(b) antigen.

### Reticulocyte Maturation

**FIGURE 11:**
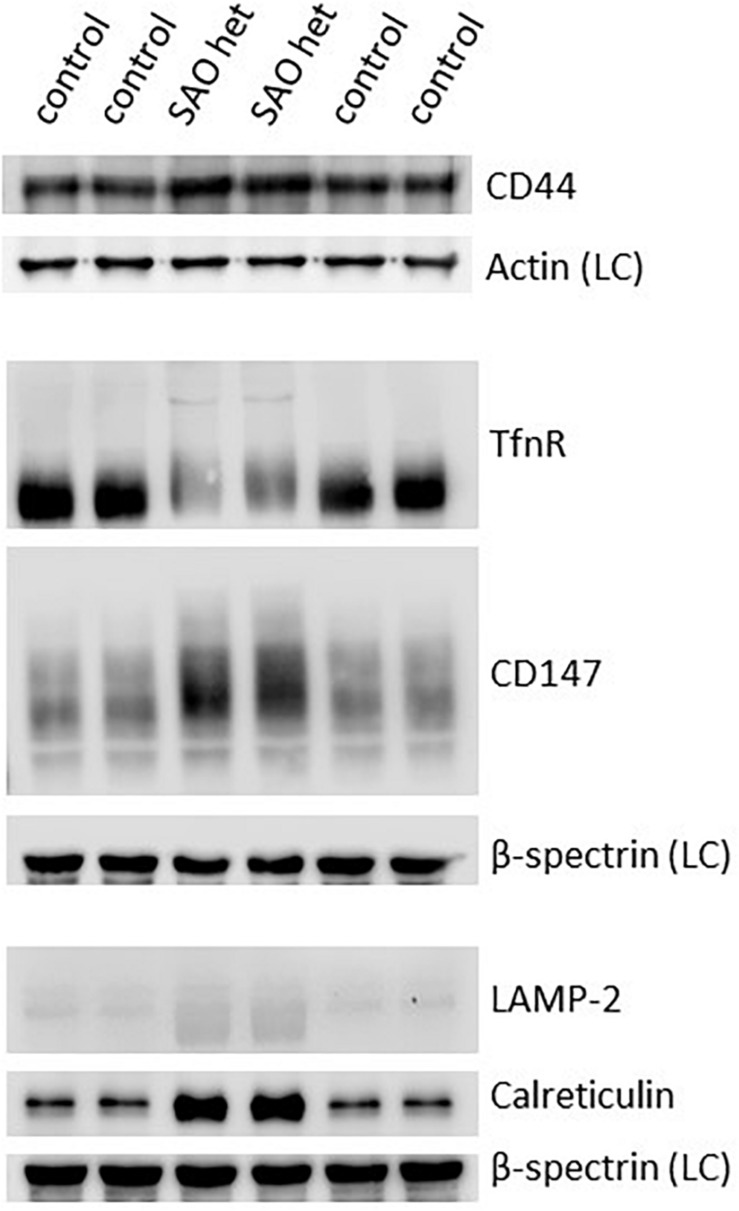
Expression of membrane proteins involved in reticulocyte maturation. Red cell membranes from heterozygous SAO and control peripheral blood samples were prepared, separated by SDS-PAGE and immunoblotted. The antibody directed against CD44 was an in-house rabbit polyclonal antibody. Commercial antibodies were used to detect actin (ab6276), calreticulin (ab2907); CD147 (ab108308), LAMP-2 (ab25631) and transferrin receptor (TfnR) (ab84036); all from Abcam, Cambridge, United Kingdom. An in-house monoclonal antibody was used to detect beta-spectrin (BRAC65). SAO het: Membranes prepared from the heterozygous father of the homozygous SAO child. LC: Loading control.

Both CD44 and CD147, proteins that would normally be reduced in the mature RBC membrane following enucleation and reticulocyte maturation, were found to be increased in the heterozygote SAO mature RBC membranes (Figure 11). CD147 also migrated more slowly than wild type in SDS-PAGE. The increase in these proteins, together with the size of mature SAO RBCs, suggested a reticulocyte maturation defect. Other proteins that would normally be depleted during reticulocyte maturation were also assessed (Figure 11). Stomatin-like protein 2 (SLP-2) and voltage dependent anion channel 1 (VDAC1), both mitochondrial proteins, were absent suggesting mitochondria are correctly cleared from SAO cells (data not shown). There was a slight increase in the level of lysosome-associated membrane protein 2 (LAMP-2) in the heterozygous SAO RBC membranes suggesting retention of some lysosomal membranes (Figure 11) and LAMP-2 had an accelerated mobility in SDS-PAGE. Levels of calreticulin, an endoplasmic reticulum (ER) protein, were much higher in the heterozygous SAO RBC membranes than in controls (Figure 11) suggesting significant retention of ER membranes. Unexpectedly transferrin receptor was substantially decreased (Figure 11). It is usual for the transferrin receptor (TfnR, CD71) to be partially lost during reticulocyte maturation. If there was a maturation defect, TfnR would be expected to remain high. However, it can be seen that the migration of TfnR in the SDS-PAGE gel was slower suggesting a trafficking defect (Figure 11) and TfnR formed internal aggregates in erythroblasts partially overlapping with the band 3 aggregates (Figure 7). So it is likely that some TfnR is degraded in the erythroblast before enucleation and reticulocyte maturation, explaining the reduced amount found in the mature SAO RBC.

## Discussion

### Dyserythropoiesis in Homozygous SAO

It is of great interest that the SAO mutation in band 3 results in a cytokinesis defect. There are two possible mechanisms; that the large amount of misfolded protein congests the system, or the intriguing possibility that SAO band 3 disrupts cytokinesis specifically. It has previously been observed that a deficiency of band 3 causes dyserythropoiesis in zebrafish (27% binuclear erythroblasts), which could be rescued by wild type band 3. Ablating the protein 4.1 binding site on band 3 prevented rescue, suggesting that the band 3-protein 4.1 interaction is critical for completion of mitosis (Paw et al., 2003). However, 2 different strains of band 3 deficient mouse models exhibited 4 and 14% erythroblast binuclearity, suggesting that other factors have a modifying effect (Bell et al., 2013). In humans, total band 3 deficiency is almost unknown and results in severe Hereditary Spherocytosis (HS). In one study of total band 3 deficiency, erythroid progenitor cells from the individual reported by Ribeiro et al. (2000) were cultured to reticulocytes without manifestation of a dyserythropoietic phenotype (Satchwell et al., 2015), although a later study of total band 3 deficiency did show a mild dyserythropoiesis (Kager et al., 2017). Therefore, the current data suggest that absence of functional band 3 does not result in a cytokinetic defect *per se*.

Consequently, the more likely explanation for the dyserythropoiesis in SAO RBCs is that the expression of large amounts of misfolded band 3 protein overwhelm the internal trafficking system of the cell. However, if this is the case then it begs the question of why dyserythropoiesis is not observed in other membrane RBC conditions where large amounts of misfolded proteins are produced. Two hereditary cation-leaky conditions, overhydrated stomatocytosis (OHSt) and stomatin-deficient cryohydrocytosis (sdCHC), are caused by mutations in non-band 3 erythrocyte membrane proteins and produce large amounts of misfolded protein but have not been associated with dyserythropoiesis (Bruce et al., 2009; Flatt et al., 2011; Bawazir et al., 2012). It has been shown that the most leaky of these, OHSt, is caused by misfolded RhAG (Bruce et al., 2009), which, like band 3, is synthesized in large quantities during erythropoiesis (∼2 × 10^5^ copies per mature RBC). A secondary effect in OHSt is the mis-trafficking of stomatin, which is retained in perinuclear vesicles, but no cytokinesis defect was seen in cultured primary erythroblasts (Fricke et al., 2005). The second most-leaky hereditary stomatocytosis condition, sdCHC, is caused by misfolded Glut1 (Flatt et al., 2011). Again, Glut1 is synthesized in large quantities during erythropoiesis (∼2–8 × 10^5^ copies per mature RBC) and stomatin is severely depleted in sdCHC RBCs (Flatt et al., 2011) but to our knowledge dyserythropoiesis has not been reported in sdCHC patients (Fricke et al., 2004; Bawazir et al., 2012).

It is notable that stomatin is absent or reduced in the above two RBC phenotypes. Stomatin is a lipid raft protein involved in RBC vesiculation (Salzer et al., 2007, 2008). There is evidence to suggest that these vesicles often contain misfolded proteins (Coleman and Hill, 2015; Leal et al., 2018) and may be a mechanism for prolonging the life span of circulating RBCs, or of stored RBCs, by removal of oxidized or damaged membrane protein. It is tempting to speculate that, during erythropoiesis stomatin may play a similar role, removing misfolded proteins by vesiculation, and that in OHSt or sdCHC erythroblasts stomatin is lost in vesicles along with the misfolded RhAG or Glut1. This would prevent accumulation of misfolded protein within the cell and congestion of the trafficking system and thus prevent dyserythropoiesis. It would also result in the depletion of stomatin. In SAO RBCs it is unlikely that the SAO band 3 can be extracted from the membrane, due to its entanglement with the cytoskeleton and other proteins, and therefore this clearance mechanism would not be available.

Overall, the evidence suggests that dyserythropoiesis in SAO RBCs is a specific effect of the expression of misfolded band 3. This conclusion is supported by a report which described another mutation in human band 3 that has been linked with dyserythropoiesis (G796R; Band 3 Ceinge; Iolascon et al., 2009). Interestingly, in this case there was also a red cell cation leak and the patient had the cryohydrocytosis (CHC) phenotype, implying the presence of misfolded band 3 at the membrane. There are numerous other band 3 mutations that give rise to CHC and where the misfolded band 3 protein is expressed, but no others have been investigated for dyserythropoiesis (Bruce et al., 2005). The CHC phenotype is very similar to the SAO phenotype, characterized by cation leaky RBCs that express a misfolded band 3 that does not transport anions or bind anion transport inhibitors (Guizouarn et al., 2011). It would be of great interest to culture CD34^+^ cells from other patients with CHC *SLC4A1* SNPs and investigate whether they too have a similar dyserythropoietic phenotype.

### Endocytic Vesicle Trafficking in SAO

It is thought that vesicle trafficking during mitosis is necessary to provide additional membrane at the site of division. Endocytosis of the plasma membrane occurs at the polar regions during anaphase and early telophase and these vesicles are then trafficked to the site of the cleavage furrow, where further endocytosis occurs during cytokinesis (Schweitzer et al., 2005). We looked for colocalization of the band 3-positive vesicles with some of the proteins implicated in this process (Arf6, clathrin) but none was observed. However, the internal band 3 vesicles colocalized with other plasma membrane proteins (Figure 8), and not with secretory pathway markers (Figure 7), suggesting endocytosis.

There was also evidence consistent with the hypothesis that large amounts of misfolded SAO band 3, trafficking from the Golgi to the plasma membrane, had overwhelmed the proteasomal degradation pathway. Such proteins can form aggregates and can be sequestered in “aggresomes,” which are subsequently subject to macroautophagy (Johnston et al., 1998). The autophagy marker, LC3, colocalized with the aggregated band 3 (Figure 7) and numerous other proteins appeared to become entangled in the aggresomes (Figures 8). Many of these proteins appeared to have been through multiple recycling loops through the ER increasing their glycan structures and apparent molecular weight (Figure 2).

In homozygous SAO cells with large aggregates it appeared that internal staining for CD71 (transferrin receptor) was increased, and was located around the aggregate (Figure 7), perhaps hinting at wider-spread disruption to vesicle trafficking in these cells. Further evidence of the wide disruption to protein movement within the SAO cells came from the analysis of proteins associating with band 3 (Figure 9). SAO band 3 had unusually strong associations with cytoskeletal proteins, spectrin, ankyrin, myosin and actin. Previous reports have shown that SAO band 3 interacts more strongly with the cytoskeleton (Mohandas et al., 1992; Sarabia et al., 1993; Liu et al., 1995). Cytoskeletal rearrangement within the erythroblast of myosin and actin was also disrupted with evidence that these proteins aggregated in clusters rather than dispersing throughout the cytoplasm as in control cells (Figure 9).

Vesicle trafficking has been shown to be important in erythroblast enucleation (Keerthivasan et al., 2010), so it is possible that disrupted vesicle trafficking contributes to the enucleation defect observed in homozygous SAO. Equally, the enucleation defect may occur because of altered band 3 associations. In WT erythroblast enucleation, a proportion of the band 3 (the mobile fraction not associated with cytoskeleton-linked complexes) distributes evenly between the pyrenocyte and the emerging reticulocyte (Lee et al., 2004). However, confocal imaging analysis of enucleation in SAO RBCs seemed to suggest that all the SAO band 3 was associated with the cytoskeleton, either through the major protein complexes, in association with ankyrin or adducin, or through simple entanglement in the cytoskeleton. The association of band 3 with GPA was also weakened, causing the loss of GPA from the reticulocyte at least in the homozygous SAO cells (Figure 10).

### Band 3 Trafficking and the Effect of SAO

Erythrocyte band 3 can be phosphorylated at tyrosine residues at positions 8, 21, 359, and 904. The phosphorylation of Tyr8 and Tyr21 is carried out by kinase Syk, which allows the phosphorylation of Tyr359 and Tyr904 by kinase Lyn (Brunati et al., 2000). It has been shown in kidney cells that phosphorylation of Tyr359 is a critical factor in band 3 trafficking (Williamson et al., 2008). In red cells, the study of a rare band 3 mutant revealed that phosphorylation of the entire protein is dependent on the presence of the first 11 amino acids (Perrotta et al., 2005). We found an increase in Tyr359 band 3 phosphorylation in homozygous SAO cells, and also saw an increase in phospho-Tyr359 in heterozygous SAO. This was striking as wild-type band 3 is only transiently tyrosine phosphorylated and longer-term tyrosine phosphorylation may only be achieved by inhibition of the highly active phosphatase using pervanadate or another protein-tyrosine phosphatase inhibitor. The SAO cells had not been treated with any phosphatase inhibitor, suggesting that band 3 Tyr359 is phosphorylated in the homeostatic state in these cells. This elevated steady-state phosphorylation of Tyr359 in SAO band 3 is another compelling indication of altered trafficking in SAO cells.

### Evidence for Oxidative Damage Caused by Misfolded SAO Band 3

PRDX2 (peroxiredoxin-2) is a cytoprotective anti-oxidant enzyme, which is upregulated in response to oxidative stress (Lee et al., 2003; De Franceschi et al., 2011). It is thought that PRDX2 associates with the membrane via binding to band 3 (Matte et al., 2013), and its membrane association is increased in cases of hereditary spherocytosis in response to oxidative stress (Rocha et al., 2008). We did not observe a significant upregulation of total PRDX2 expression during homozygous SAO erythropoiesis (Figure 6C). However, we found that PRDX2 is present at significantly higher levels in heterozygous SAO ghost membranes compared to controls (Figure 2), which is suggestive of oxidative stress in these cells. The presence of aggresomes has been linked to oxidative damage and inflammation (Luciani et al., 2010), with the consequence of further cellular damage if they are not successfully cleared. The large amounts of accumulated misfolded band 3 in SAO may therefore be the cause of oxidative damage in these cells.

### Incomplete Reticulocyte Maturation in SAO

The homozygous SAO red cells found in the circulation are large and stomatocytic. They have failed to lose their excess membrane and remodel their cytoskeleton to form a biconcave disc. In healthy cells proteins and membrane are lost at enucleation and during reticulocyte maturation (Johnston et al., 1998; Griffiths et al., 2012; Mankelow et al., 2016). The increased cytoskeletal attachment of SAO band 3 may inhibit cytoskeletal reorganization, endocytosis and vesicle expulsion required for reticulocyte maturation (Griffiths et al., 2012). We have found an altered distribution of proteins at enucleation and the presence of nuclei, organelles and proteins that should have been cleared from the cells (Figures 1B, 2, 9B, 10). CD147 is one such protein, in that the majority partitions with the extruded nucleus during enucleation and then is further depleted by removal in vesicles during reticulocyte maturation (Griffiths et al., 2012). In homozygous SAO late erythroblasts there are normal levels of CD147 but increased amounts in mature red cells (and in heterozygote red cells), suggestive of a defect in reticulocyte maturation. CD44 is usually quantitatively reduced during the late stages of erythropoiesis, but also remains high in cells expressing SAO band 3. Incomplete clearance of organelles from SAO cells was shown by the increased amounts of LAMP-2 (lysosome) and calreticulin (ER), although clearance of mitochondria appeared to be normal.

### Similarities Between Dyserythropoiesis in SAO and CDAII Cells

In both SAO and CDAII erythroblasts there is a cytokinesis defect, the cells fail to separate and there are numerous binuclear cells. Immunogold electron microscopy of CDAII cells detects a dense discontinuous ring of ER-derived membrane beneath the plasma membrane (Alloisio et al., 1996). In fact, the authors suggest that increased levels of glucose-regulated protein GRP78, calreticulin or protein disulfide isomerase (PDI), all major protein of the ER, could be used as markers for CDAII (Alloisio et al., 1996). A similar analysis of SAO membranes, using electron microscopy, has not been done but we have shown a massive increase in calreticulin in heterozygous SAO membranes (Figure 2) suggesting that the SAO cells still contain significant amounts of ER membranes, although it is also possible that calreticulin is expressed in the plasma membrane of SAO cells. The immunoprecipitation of homozygous erythroblast lysates also showed a strong association between band 3 and HSP70, otherwise known as GRP78, an ER-associated heat shock protein (Figure 9). Another protein that has been suggested as a marker for CDAII is CD44 (Singleton et al., 2018) and we found that CD44 protein is high in SAO RBCs. One difference between SAO and CDAII is the apparent molecular weight of the band 3 protein when separated by SDS-PAGE. SAO band 3 migrates slower than normal whereas CDAII band 3 has a faster mobility (Denecke et al., 2008). Nonetheless, it would be of interest to investigate the expression of band 3 through erythropoiesis in CDAII cells. As band 3 is the major membrane protein expressed in RBC, with 10^6^ copies per RBC, the Sec23b mutation in CDAII cells that causes a trafficking defect is quite likely to result in the accumulation of band 3 aggregates similar to those seen in SAO cells.

### Clinical Significance of the Heterozygous SAO Mutation

Homozygous SAO is an extremely rare condition as it is usually lethal (Mgone et al., 1996), however, heterozygous SAO is relatively common in certain parts of SE Asia and Papua New Guinea or other regions of the world where malaria is rife (Amato and Booth, 1977; Mgone et al., 1996; Paquette et al., 2015). A greater understanding of the SAO phenotype is useful for the clinical care of individuals with SAO. One study showed that babies with heterozygous SAO often have a significant anemia at birth due to hemolysis, and 16 of the 31 babies studied developed neonatal hyperbilirubinemia, but this reverts to normal in the first few months of life (Laosombat et al., 2010). Although heterozygous SAO is apparently asymptomatic in adults, we observed a multinuclear phenotype in cultured heterozygous SAO erythroblasts (albeit to a lesser degree than homozygous erythroblasts), altered protein trafficking and impaired reticulocyte maturation and changes in the membrane protein composition of mature heterozygous SAO RBCs including oxidative damage. So it is possible that individuals with heterozygous SAO could experience complications when presenting with other conditions. For example, as band 3 SAO does not transport anions, inheritance of band 3 SAO *in trans* to another mutant band 3 allele such as one causing the loss of band 3 and hereditary spherocytosis can result in severe hemolytic anemia and dRTA (Alexander et al., 2019).

## Concluding Remarks

In this study we examined in greater depth the phenotype of the first known case of homozygous SAO. Despite the absence of wild type band 3, SAO band 3 is expressed at the red cell surface. During *in vitro* culture, erythroid progenitors from the proband exhibited a defect in the late phase of cell division, cytokinesis, resulting in multinucleated cells, which occurred along with a clear reduction in enucleation. Culture of the parents’ heterozygous SAO erythroid progenitor cells produced a milder version of this dyserythropoietic phenotype. This study has shown that the production of misfolded SAO band 3 overwhelms the usual protein quality control mechanisms and results in the formation of large areas of aggregated protein in some cells. These colocalized with the autophagosomal marker LC3.

The misfolded SAO band 3 was altered in its reactivity with monoclonal anti-band 3 antibodies, and immunoprecipitation experiments supported previous findings that SAO band 3 associates more strongly with cytoskeletal proteins. A strong interaction between SAO band 3 and non-muscle myosins IIA and IIB was detected, along with perturbed cellular localization of myosin IIA and actin, supporting the hypothesis that the important role of myosin in cytokinesis is blocked and results in the dyserythropoietic phenotype.

## Data Availability Statement

All datasets generated for this study are freely available from the corresponding author.

## Ethics Statement

The studies involving human participants were reviewed and approved by National Health Service National Research Ethics Service South West. Written informed consent to participate in this study was provided by the participants’ legal guardian/next of kin.

## Author Contributions

JF designed and performed experiments, analyzed data, and wrote the manuscript. NC performed culture experiments. CS-H, DE, and NH performed immunoblotting experiments. KH conducted mass spectrometry experiments. VP and CT provided patient samples and edited the manuscript. LB designed the study, analyzed data, and edited the manuscript.

## Conflict of Interest

The authors declare that the research was conducted in the absence of any commercial or financial relationships that could be construed as a potential conflict of interest.

## Footnotes

1 http://imagej.nih.gov/ij/
